# Larvicidal Activity of Hemp Extracts and Cannabidiol against the Yellow Fever Mosquito *Aedes aegypti*

**DOI:** 10.3390/insects15070517

**Published:** 2024-07-10

**Authors:** Erick J. Martínez Rodríguez, P. Larry Phelan, Luis Canas, Nuris Acosta, Harinantenaina L. Rakotondraibe, Peter M. Piermarini

**Affiliations:** 1 College of Food, Agriculture and Environmental Sciences, The Ohio State University, Wooster, OH 44691, USA; martinezrodriguez.2@buckeyemail.osu.edu (E.J.M.R.); phelan.2@osu.edu (P.L.P.); canas.4@osu.edu (L.C.); acosta.26@osu.edu (N.A.); 2 College of Pharmacy, The Ohio State University, Columbus, OH 43210, USA; rakotondraibe.1@osu.edu

**Keywords:** hemp, extracts, cannabidiol (CBD), mosquito, larvicide, insecticide resistance

## Abstract

**Simple Summary:**

The present study examined whether extracts of hemp leaves were toxic to *Aedes aegypti* larvae and determined which compound(s) were responsible for the toxicity. We found that larvae, from both insecticide-resistant and -susceptible strains were killed by hemp leaf extract within 48 h of exposure. Furthermore, we found that an abundant cannabinoid (cannabidiol) within the extract was the primary active compound. This study suggests that hemp extracts and cannabidiol are potentially valuable sources for developing biopesticides to control mosquitoes.

**Abstract:**

To mitigate pyrethroid resistance in mosquito vectors of emerging and re-emerging human pathogens, there is an urgent need to discover insecticides with novel modes of action. Natural alternatives, such as extracts derived from plants, may serve as substitutes for traditional synthetic insecticides if they prove to be sustainable, cost-effective, and safe for non-target organisms. Hemp (*Cannabis sativa*) is a sustainable plant known to produce various secondary metabolites with insecticidal properties, including terpenoids and flavonoids. The goal of this study was to assess the larvicidal activity of hemp leaf extract on mosquito larvae from both pyrethroid-susceptible (PS) and pyrethroid-resistant (PR) strains of *Aedes aegypti*. Another goal was to identify which components of the extract were responsible for any observed larvicidal activity. We found that a methanol extract of hemp leaves induced similar concentration-dependent larvicidal activity against PS (LC_50_: 4.4 ppm) and PR (LC_50_: 4.3 ppm) strains within 48 h. Partitioning of the leaf extract between methanol and hexane fractions revealed that full larvicidal activity was restricted to the methanol fraction. Analysis of this fraction by gas chromatography–mass spectrometry and nuclear magnetic resonance showed it to be dominated by cannabidiol (CBD). Larvicidal assays using authentic CBD confirmed this compound was primarily responsible for the toxicity of the hemp leaf extract against both strains. We conclude that hemp leaf extracts and CBD have the potential to serve as viable sources for the development of novel mosquito larvicides.

## 1. Introduction

Mosquitoes are considered the most dangerous animals on Earth because they are vectors of numerous pathogens that cause deadly and debilitating diseases in humans and domestic animals, including malaria, West Nile virus, and heartworm [1,2,3,4]. The yellow fever mosquito *Aedes aegypti* is a vector of several arboviruses of medical importance, including chikungunya, dengue, yellow fever, and Zika. To limit the transmission of mosquito-borne arboviruses, humans have relied heavily on insecticides to control the larval and/or adult life stages of mosquitoes. Synthetic insecticides, such as pyrethroids, are critical chemical tools used for control; however, the widespread use of insecticides with limited modes of action has led to resistance [5,6,7]. Additionally, the overuse of these insecticides has led to detrimental impacts on both human and environmental health [8]. Thus, the discovery of alternatives to synthetic pesticides, like biopesticides, is crucial to improving mosquito management.

Plant secondary metabolites are considered a valuable resource for the discovery of bioactive compounds that can be developed into novel biopesticides [9,10,11,12]. The hemp plant, *Cannabis sativa*, produces more than 1000 different secondary metabolites, including highly volatile and aromatic terpenes and phenols, as well as semi-volatile phytocannabinoids [13]. Notably, the terpenes linalool, eucalyptol, p-cymene, and thymol have shown larvicidal and/or adulticidal activity in various mosquito species [14,15,16]. Furthermore, phytocannabinoids possess insecticidal and/or antifeedant activity against herbivorous insects. For example, tobacco hornworm larvae (*Manduca sexta*) preferred feeding on hemp leaf tissue containing lower vs. higher cannabidiol (CBD) concentrations [17]. In addition, cabbage looper larvae (*Trichoplusia ni)* consumed more leaf area in cannabinoid-free genotypes than in cannabinoid-dominant genotypes [18]. Notably, larval survival and growth of *T. ni* were reduced when cannabidiolic acid (CBDA) and cannabigerolic acid (CBGA) were incorporated into larval diets [18]. Moreover, cannabidiol (CBD) oil (3%) impairs development and lowers survival rates in stored product insect pests (*Plodia interpunctella*, *Oryzaephilus surinamensis*, and *Tribolium confusum*) [19]. Thus, the hemp plant produces a variety of secondary metabolites with potential for mosquito control.

Consistent with this notion, hemp-based essential oils and extracts are larvicidal and adulticidal against multiple mosquito species [20,21,22,23,24,25]. However, the cross-resistance of pyrethroid-resistant mosquito strains to hemp extracts and the specific compound(s) responsible for mosquitocidal activities within the extracts are unknown. To address these gaps in knowledge, the objectives of this study were to (1) compare the larvicidal potency of hemp leaf extracts against pyrethroid-susceptible (PS) and pyrethroid-resistant (PR) strains of *Ae. aegypti* and (2) identify the principal active larvicidal ingredients in the extracts that elicit mortality.

## 2. Materials and Methods

### 2.1. Hemp Plants

The hemp material used to obtain the raw extract was grown under controlled greenhouse conditions (24 °C 14:10 L:D cycle). The cuttings for the new plants used for the experiment were obtained from previously grown hemp mother plants (variety Tango Kush). In brief, fresh shoots were cut using a sanitized razor blade, and the cuttings were placed in a 500 ppm DIP ‘N’ Grow Hormonal Rooting Concentrate (Clackamas, OR, USA) for 30 s. The cuttings were then transferred to a 34 ct double strip OASIS wedge (Grow It Depot, Long Branch, NJ, USA) filled with Pro-Mix BX soilless medium (Premier Tech, Rivière-du-Loup, QC, Canada) and placed in a mist chamber at controlled conditions. When root growth was adequate, hemp plants were transplanted into 38 L plastic pots and placed in a greenhouse room maintained at 25 °C, 16:8 (L:D), by an Argus Control System (Conviron, Langley Twp, BC, Canada).

### 2.2. Hemp Extract

Leaves were removed from the hemp plants 7 months after planting and air-dried at 25 °C for 7 days. Dried vegetative material was pulverized for 5 min using a coffee grinder; 150 g of powdered material was extracted in 4 L of methanol for 3 weeks at 20 °C with daily shaking for 2–3 min. The incubated solution was then filtered through a membrane filter paper on top of a Porcelain Buchner funnel (Fisherbrand, Waltham, MA, USA) attached to a Little Giant pressure vacuum pump (Gelman Instrument Company, Ann Arbor, MI, USA). The solution was placed in a Hei-Vap Rotory Evaporator (Heidolph NA, Wood Dale, IL, USA) at 30 °C and 65 rpm to remove the methanol. The resulting crude residue (dried leaf extract) was kept at 4 °C, and aliquots were resuspended in 100% acetone immediately prior to use in bioassays.

### 2.3. Methanol and Hexane Partitioning

We partitioned 50 mg of dried extract between 20 mL hexane and 20 mL methanol using a 60 mL separatory funnel (Chemglass Life Sciences LLC, Vineland, NJ, USA), with the resulting partitions collected into separate 40 mL glass vials (Thermo Scientific, Rockwood, TN, USA). The solutions were then evaporated to dryness under nitrogen at room temperature, using a Reacti-Vap (Thermo Scientific, Waltham, MA, USA) connected to a Nitrox UHPLCMS12 nitrogen generator (Domnick Hunter, Gateshead, UK). The residues were resuspended in acetone to reach the desired concentration for mosquito bioassays or gas chromatography/nuclear magnetic resonance analysis.

### 2.4. Gas Chromatography–Mass Spectrometry (GC-MS) and Nuclear Magnetic Resonance (NMR)

GC-MS analyses were performed using an Agilent Technologies 7890A GC equipped with a 7683B auto-sampler and interfaced to a 5975C inert mass selective detector (Agilent Technologies, Santa Clara, CA, USA). A 1 µL sample was injected splitless at 280 °C, with a constant He flow of 1.1 mL/min. The GC column was HP-5MS, 30 m × 250 µm diam × 0.25 µm film thickness. The oven was programmed with an initial temperature of 35 °C for 1 min, increased 7 °C/min to 100 °C, then 25 °C/min to 280 °C and held for 10 min, with the MS transfer line at 280 °C. The MS was operated in scan mode using *m*/*z* 19–450 with source at 230 °C and quadrupole at 150 °C. GC peaks were identified by searching their mass spectra against the NIST/EPA/NIH Mass Spectral Library and comparing relative retention times against published values. To quantitate CBD, a calibration curve was prepared from a CBD isolate (99%, Extract Labs, Boulder, CO) dissolved in acetone and injected into the GC-MS (19.4–525 ng, linear R^2^ = 0.98).

Hemp extracts and methanol/hexane partitions were also analyzed via proton NMR. They were first dissolved in 0.6 mL of deuterated chloroform before analysis with a Bruker AVANCE III 400 MHz NMR (Bruker, Billerica, MA, USA). Resonances were analyzed and compared with previously published data [26].

### 2.5. Aedes Aegypti Colonies and Strains

Larvae of *Ae. aegypti* from Liverpool (strain LVP-IB12, MRA-735, contributed by David W. Severson) and Puerto Rico (strain Puerto Rico, NR-48830, contributed by G.G. Clark & J.J. Becnel) strains were reared from eggs using established methods [27,28]. The original eggs were provided by the Centers for Disease Control and Prevention for distribution by BEI Resources, NIAID, NIH. Larvae of the Puerto Rico strain used in the present study were over 30 times more resistant to the pyrethroid cypermethrin compared to the Liverpool strain (Supplemental Figure S1). From here, we refer to the Liverpool strain as pyrethroid-susceptible (PS) and the Puerto Rico strain as pyrethroid-resistant (PR). Larvae from both strains were nourished with 1 tablet per day of fish food (Tropical Tablets, Tetramin, Blacksburg, VA, USA). Adult mosquitoes were provided with 10% sucrose solution ad libitum. For additional egg production, adult females were fed defibrinated rabbit blood (Hemostat Laboratories, Dixon, CA, USA) for a period of 2 h, using a membrane feeder (Hemotek, Blackburn, UK). All mosquitoes were reared in environmentally controlled chambers held at 28 °C and 80% RH, with a 12:12 L:D cycle.

### 2.6. Larval Bioassay

Larvicidal activities of hemp extracts, methanol/hexane partitions, and CBD isolates were determined using an established high-throughput bioassay [28,29], which follows the World Health Organization (WHO) guidelines. In brief, to each well of a 24-well Falcon^®^ Multiwell plate (Becton Dickinson Labware, Franklin Lakes, NJ, USA), the following was added: 985 µL of deionized H_2_O (diH_2_O), six 1st-instar *Ae. aegypti*, 5 µL of food solution (13 mg/mL of finely ground fish food flakes; Tetramin, Blacksburg, VA, USA), and 10 µL of a treatment (hemp extract, methanol fraction, hexane fraction, CBD isolate, or suitable solvent control). The hemp extract and the methanol and hexane fractions were diluted serially with acetone to 100, 33, 11, 3.6, and 1.2 ppm. The CBD isolate was diluted serially with acetone to 20, 6.0, 2.2, 0.7, and 0.24 ppm. The CBD isolate concentrations were selected to match the corresponding CBD concentrations in the hemp extracts as determined by GC-MS. The bioassay plates were held under standard rearing conditions. Larvae were considered dead if they did not move after gently touching their abdomen with a fine needle or pipette tip [29]. Mortality was assessed at 48 h because at 24 h, we detected sublethal effects (e.g., slow-moving or twitching larvae) at intermediate concentrations that introduced subjectiveness into the mortality assessment. All mortality values were corrected for solvent control mortality using Abbott’s formula [30]. If solvent control mortality exceeded 20%, then the trial was excluded.

### 2.7. Statistical Analysis

Data analysis and plotting were performed using GraphPad Prism (version 6.07) software (GraphPad, San Diego, CA, USA). Median lethal concentrations (LC_50_) were determined by plotting percent mortalities against log transformations of the concentration tested. A non-linear regression (‘log(agonist) vs. normalized response’ function) was used to best fit the data and calculate LC_50_. Statistical comparisons of LC_50_ values were performed through sum-of-squares F-tests (α = 0.05).

## 3. Results

### 3.1. Hemp Leaf Extract Toxicity against Larvae

**Figure 1:**
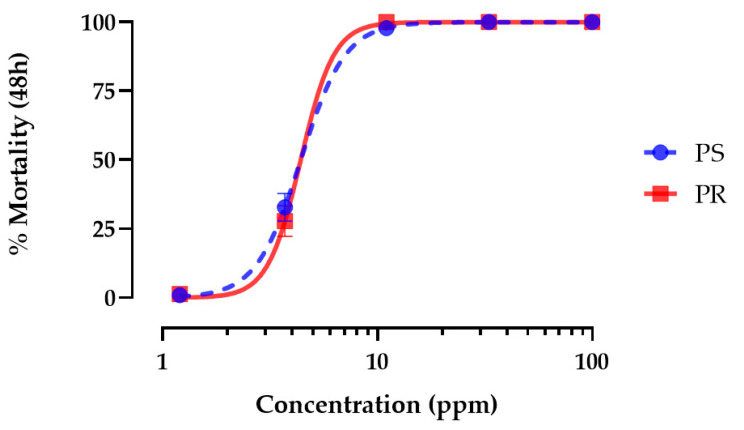
Concentration–response curves for 48 h larvicidal activity of hemp leaf extract against pyrethroid-susceptible (PS, blue) and pyrethroid-resistant (PR, red) *Ae. aegypti*. Values plotted are means ± standard errors of the mean (SEM) based on 28 replicates of 6 larvae per concentration (1.2, 3.7, 11, 33, 100 ppm).

Application of hemp leaf extracts to the rearing water of first-instar *Ae. aegypti* caused concentration-dependent mortality in both PS and PR strains within 48 h that reached 100% (Figure 1). The LC_50_ value of leaf extracts in the PS strain (4.4 ppm; 95% CI = 4.0–4.8 ppm) was not different (*p* = 0.85; F = 0.03) than that of the PR strain (4.3 ppm; 95% CI = 2.37–7.70 ppm).

### 3.2. Methanol and Hexane Partition Toxicity against Larvae

**Figure 2:**
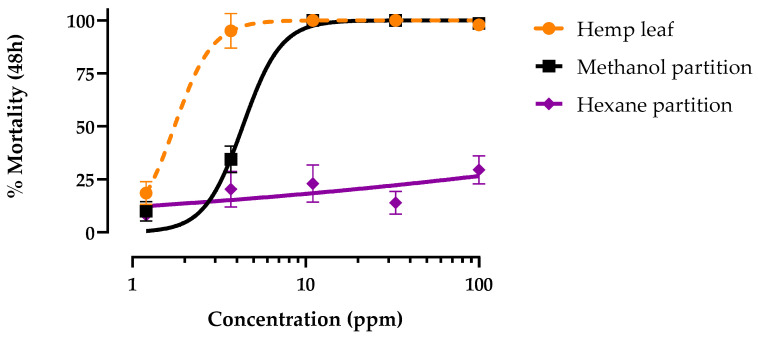
Concentration–response curves for 48 h larvicidal activity of methanol (black) and hexane (purple) partitions of hemp leaf extract (orange) against pyrethroid-susceptible *Ae. aegypti*. Values are means ± SEM based on 12 replicates of 6 larvae per concentration (1.2, 3.7, 11, 33, and 100 ppm).

Hemp leaf extract was partitioned between methanol and hexane to separate polar and nonpolar constituents, respectively. These partitions were then tested in parallel with the original unpartitioned hemp leaf extract for larvicidal activity against the PS strain. Both the methanol partition and unfractionated leaf extract produced concentration-dependent mortality within 48 h that reached 100% (Figure 2). However, the LC_50_ of the methanol partition (4.3 ppm; 95% CI = 3.8–4.9 ppm) was ~2.5 times less potent (*p* < 0.001, F = 88.1) than that of the unfractionated leaf extract (1.7 ppm; 95% CI = 1.4–2.0 ppm). In contrast, the hexane fraction did not elicit concentration-dependent mortality, reaching only 25% mortality at the maximal concentration tested (Figure 2).

### 3.3. GC-MS and NMR Analysis

**Figure 3:**
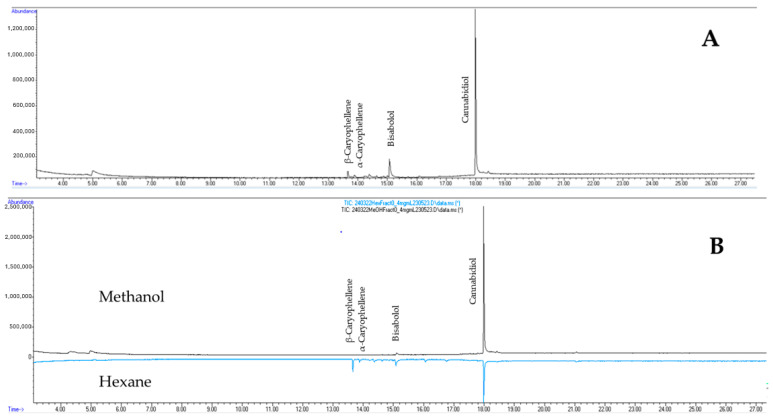
Gas chromatogram of (**A**) hemp leaf extract and (**B**) methanol partition (black) with hexane partition (blue) inverted.

GC-MS identified CBD as the most abundant compound in the original hemp leaf extract (Figure 3A) and both partitions (Figure 3B); however, the methanol partition contained ~4× more CBD (80% of CBD found in leaf extract) compared to the hexane partition (20%) (Figure 3B). Additionally, the analysis indicated the presence of other compounds, like α- and β-caryophellene and bisabolol, in both the unpartitioned extract and hexane partition but not the methanol partition (Supplemental Table S1). The ^1^H NMR spectra of the leaf extracts measured in deuterated chloroform were superposable with the ^1^H NMR spectrum reported by Barthlott et al. [26]. Since CBD was observed as the major compound, a quick ^13^C NMR was collected to confirm its presence in the extract (Supplemental Figure S2). Moreover, signals arising from proton (^1^H) of the active methanol partition showed CBD as the major compound and a trace of tetrahydrocannabidiol (THC) analogs (Supplemental Figure S3). Resonances arising from the protons of bisabolol were also observed (δ 5.36, brs; δ 5.12, brt; and singlet methyls at δ ~1.6 ppm; Supplemental Figure S4); they were similar to those reported by Cerceau et al. [31].

### 3.4. CBD Toxicity against First-Instar Larvae

**Figure 4:**
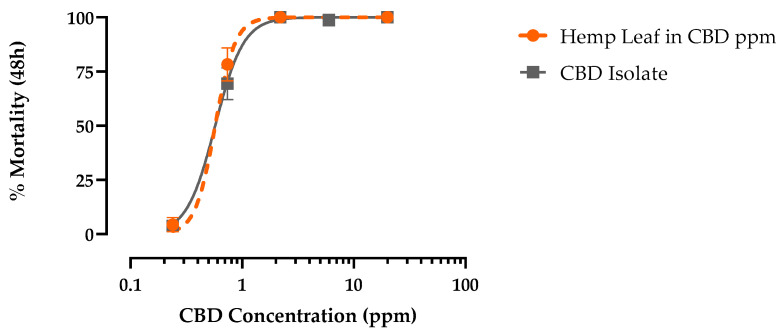
Concentration–response curves for 48 h larvicidal activity of CBD (grey) against pyrethroid-susceptible *Ae. aegypti*. Values plotted are means ± SEM based on 16 replicates of 6 larvae per concentration (0.2, 0.7, 2.2, 6.0, and 20 ppm).

**Figure 5:**
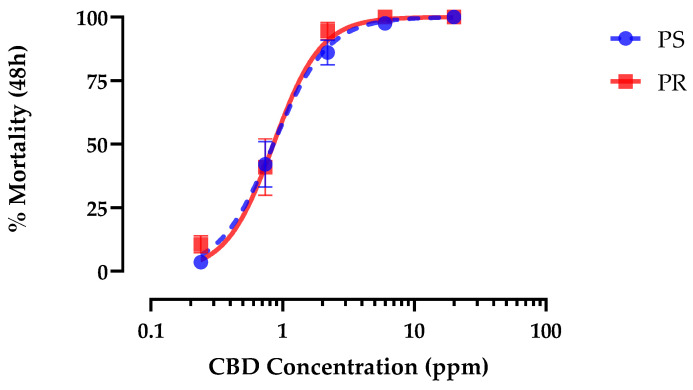
Concentration–response curves for 48 h larvicidal activity of CBD against pyrethroid-susceptible (PS, blue) and pyrethroid-resistant (PR, red) *Ae. aegypti*. Values are means ± SEM based on 16 replicates of 6 larvae per concentration (0.2, 0.7, 2.2, 6.0, and 20 ppm).

Authentic CBD produced concentration-dependent mortality in PS first-instar larvae within 48 h that reached 100% and was indistinguishable from hemp leaf extract when standardized for CBD concentration (Figure 4). The LC_50_ of CBD (0.59 ppm, 95% CI = 0.52–0.67 ppm) was similar (F = 0.95, *p* > 0.32) to hemp leaf extract (0.53 ppm, 95% CI = 0.46–0.62 ppm) in PS first-instar *Ae. aegypti* (Figure 4). Moreover, as found for the hemp leaf extract, CBD elicited similar (F = 0.26, *p* > 0.6) larvicidal potency against PR (LC_50_ = 0.83 ppm, 95% CI = 0.69–0.99 ppm) and PS (LC_50_ = 0.88 ppm, 95% CI = 0.75–1.04 ppm) strains (Figure 5).

## 4. Discussion

We demonstrated that hemp leaf extracts elicit concentration-dependent larvicidal activity against *Ae. aegypti*. These results are consistent with previous studies that have found the concentration-dependent larvicidal activity of hemp extracts against other mosquitoes, including *Culex quinquefasciatus*, *Anopheles stephensi*, *An. gambiae*, *Ae. albopictus*, and *Ae. aegypti* [16,20,21,22,23,32]. In addition, we demonstrated that hemp leaf extracts exhibited a similar toxic potency against a PR strain of *Ae. aegypti* that has both target-site (*kdr*) and metabolic resistance to pyrethroids [5,7,33,34]. Our finding suggests that hemp leaf extracts have the potential to bypass pyrethroid resistance in mosquito larvae.

We also identified CBD as the primary active ingredient within the hemp extract responsible for its larvicidal activity. CBD was by far the most abundant compound in the hemp leaf extracts and methanol partitions, as detected by GC-MS and proton NMR. Terpenes, which have previously been speculated as the primary larvicidal compounds in hemp extracts [24,35,36,37,38,39], were of nominal abundance. Moreover, the hexane fraction of the hemp extract, in which terpenes are expected to partition, was minimally larvicidal. Importantly, the larvicidal potency of a CBD isolate matched that of the hemp leaf extract in both the PS and PR strains of *Ae. aegypti*. Moreover, the larvicidal potency of the methanol partition of the leaf extract was slightly weaker than that of the unpartitioned hemp leaf extract but far superior to that of the hexane partition of the leaf extract, consistent with the relative abundances of CBD in these samples.

Our findings regarding the toxicity of CBD to mosquitoes align well with prior research suggesting CBD has toxic, antifeedant, and/or growth-inhibiting properties against other insects. For example, the larvae of three economically important lepidopteran pests, the tobacco hornworm (*M. sexta*), the corn earworm (*Helicoverpa zea*), and the fall armyworm (*Spodoptera frugiperda*), all showed reduced size, weight loss, and decreased consumption rates when feeding on diets supplemented with CBD [17,40,41]; additionally, *M. sexta* larvae that consumed high doses of CBD experienced higher mortality [17]. Likewise, larvae of the cabbage looper, *T. ni*, consumed less leaf area on CBD-dominant *C. sativa* genotypes compared to the cannabinoid-free genotypes, leading to a decrease in larval mass and an increase in mortality [18]. Furthermore, in larvae of three common stored product insect pests, the meal moth (*P. interpunctella*), saw-toothed grain beetle (*O. surinamensis*), and flour beetle (*T. confusum*), mortality significantly increased after exposure to grains that had been sprayed with high doses of CBD oil [19].

The specific mode of action of CBD toxicity against mosquitoes and other insects is unknown. Intriguingly, insects are one of the few animal groups that do not possess canonical cannabinoid receptors [42,43]. However, at least in mammalian systems, CBD is known to modulate a wide range of biochemical targets, including orthologs of known insecticide targets, such as sodium channels, potassium channels, calcium channels, transient receptor potential channels, G protein-coupled receptors, and acetylcholinesterase [44,45,46,47,48]. Thus, CBD likely affects multiple biochemical targets and tissues in insects. Notably, in the ventral chain ganglia of *M. sexta* larvae, CBD treatment delayed the onset of electrophysiological responses to electrical stimuli, but the magnitude of the responses was enhanced [17]. Whether these neuromodulatory effects of CBD contribute to its toxicity in insects remains to be determined.

Altogether, our study provides additional evidence supporting the notion that hemp is a valuable potential resource for developing novel insecticides to control mosquitoes [21,32,49]. The promising results of the present study motivate future studies to further evaluate hemp extracts and CBD as potential larvicides as well as to determine potential non-target and environmental impacts of using hemp extracts and CBD as larvicides. In addition, future studies should evaluate the economic feasibility of using hemp leaves as a source of insecticides. Notably, hemp is an emerging, readily cultivated crop in the U.S. [50,51,52,53], and its leaves are often discarded [21,49]. Thus, the availability of raw materials would not appear to be a limiting factor as it can be for other sources of biopesticides [35]. Lastly, it should be emphasized that we only examined one strain of hemp in the current study. Numerous strains of hemp varieties are available with highly diverse secondary metabolite profiles [13,54,55]. Future studies should screen various hemp strains to identify additional active ingredients besides CBD and determine which strains would be most efficient for the cultivation of hemp for biopesticide production.

## 5. Conclusions

The present research has shown that hemp leaf extracts have toxic larvicidal activity against pyrethroid-susceptible PS and pyrethroid-resistant PR strains of the yellow fever mosquito *Ae. aegypti*. Moreover, cannabidiol (CBD) appears to be the principal active ingredient responsible for larvicidal activity.

## Data Availability

Data are contained within the article or Supplementary Material.

## Supplementary Materials

The following supporting information can be downloaded at: https://www.mdpi.com/article/10.3390/insects15070517/s1, Table S1: Compounds identified in GC/-MS analysis of hemp leaf extract and its hexane and methanol fractions. The retention time (R.T.) indicates the minutes in which compounds were detected. All listed compounds were present in the hemp leaf extract (+). The values for the hexane and methanol fractions represent the percentage (%) of each compound relative to its total amount in the hemp leaf extract; Figure S1: Concentration–response curves for 48 h larvicidal activity of Cypermethrin against *Ae. aegypti* of pyrethroid-susceptible (PS, blue) and pyrethroid-resistant (PS, red) strains. Values are means ± SEM based on 8 replicates of 6 larvae per concentration (0.001, 0.01, 1, and 100 ppm); Figure S2: Blue: ^1^H NMR spectrum of dried hemp leaf extract with signals between 6.10 and 6.39 ppm expanded. Black: expansion of signals between 6.05 and 6.45 ppm that shows the characteristic signals of CBD and THC derivatives as shown in Bartlott et al. [27]; Figure S3: ^13^C NMR of dried leaf/unfractionated leaf extract showing the presence of CBD as major compound; Figure S4: NMR spectra of the hexane and methanol partitions of hemp leaf extract showing the presence of CBD and bisabolol as major and minor compounds, respectively.
