# Supplementary material for: Larvicidal Activity of Hemp Extracts and Cannabidiol against the Yellow Fever Mosquito Aedes aegypti
Source: Insects. 2024 Jul 10;15(7):517. doi: 10.3390/insects15070517 (PMC11276947; doi:10.3390/insects15070517)
Supplement: Supplementary file 1 [file insects-15-00517-s001.zip › insects-3016633-supplementary.pdf]

Supplemental Table S1. Compounds identified in GC/-MS analysis of hemp leaf dried extract and its hexane and methanol fractions. The retention time (R.T.) indicates the minutes at which compounds were detected. All listed compounds were present in the dried hemp leaf extract (+). The values for the hexane and methanol fractions represent the percentage (%) of each compound relative to its total amount in the dried leaf extract.

Compound	R.T. (min)		Percentage (%)	
		Hemp leaf dried extract	Hexane fraction	Methanol Fraction
β-Caryophellene	13.65	+	100	0
α-Caryophellene	13.81	+	100	0
Bisabolol	15.06	+	90	10
CBD	17.98	+	20	80

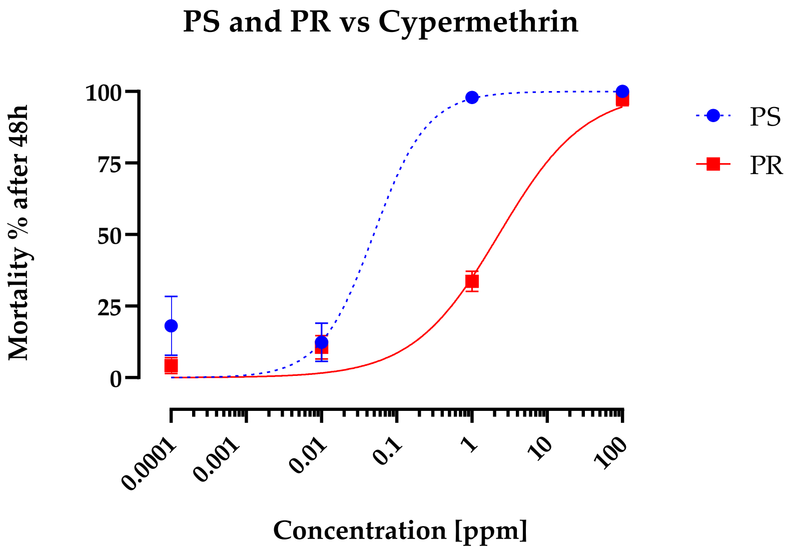

Supplemental Figure S1. Concentration-response curves for 48 h larvicidal activity of cypermethrin against pyrethroid-susceptible *Ae. aegypti* of (PS, **blue**) and pyrethroid-resistant (PR, **red**) strains of *Ae. aegypti*. Values are means ± SEM based on 8 replicates of 6 larvae per concentration (0.001, 0.01, 1, and 100 ppm).

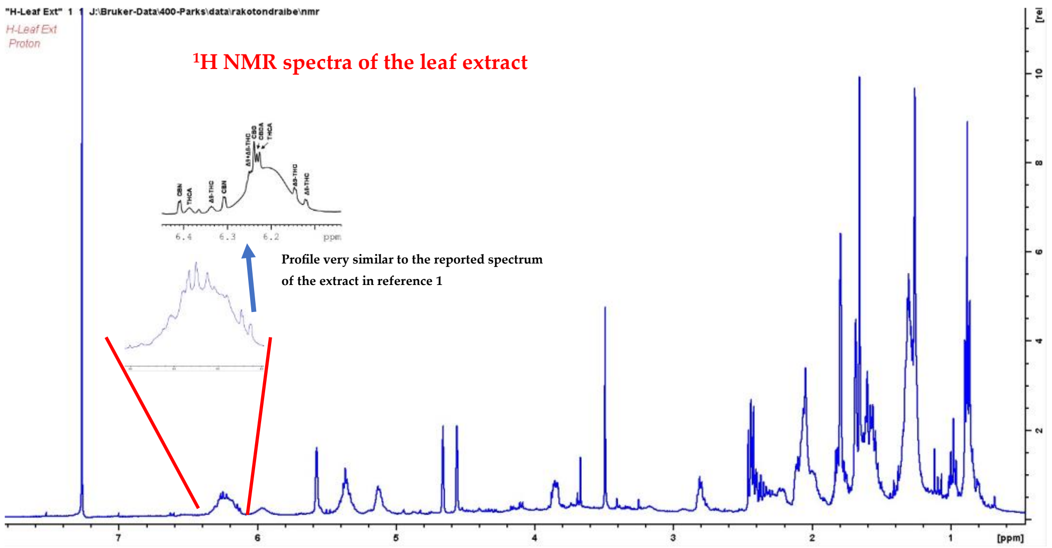

Supplemental Figure S2. ^{1}H NMR spectra of dried hemp leaf extract

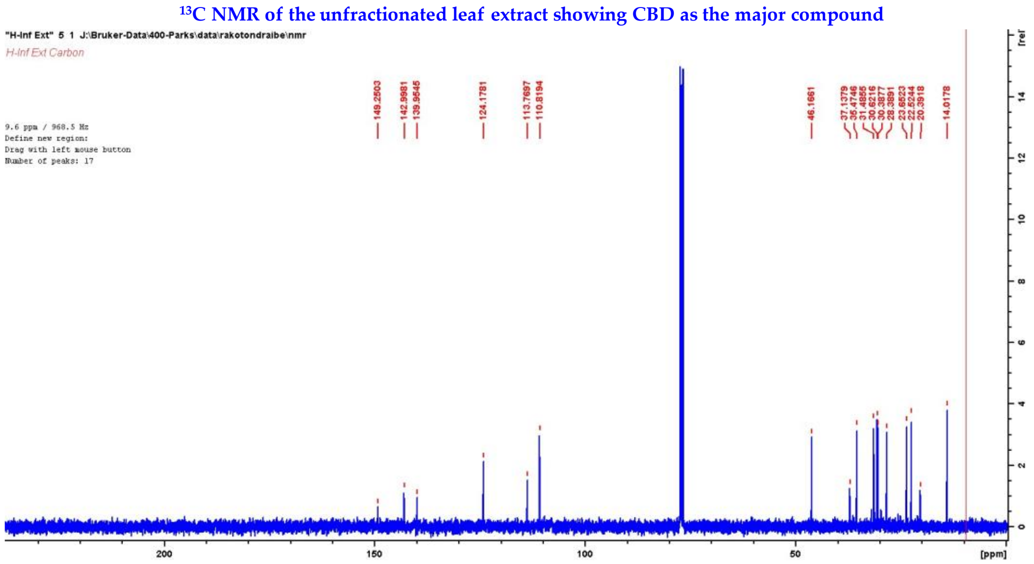

Supplemental Figure S3. ^{13}C NMR of dried leaf/unfractionated leaf extract.

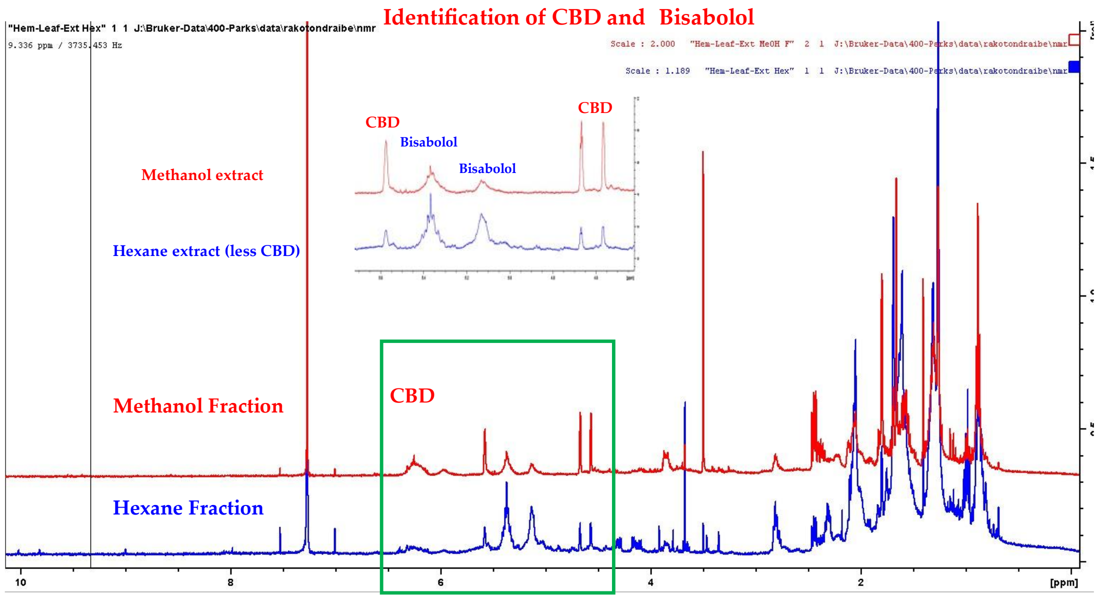

Supplemental Figure S4. NMR analysis of CBD and bisabolol found in methanol and hexane fractions of unfractionated dried hemp leaf extract.
